# Scattering Coefficients of Mice Organs Categorized Pathologically by Spectral Domain Optical Coherence Tomography

**DOI:** 10.1155/2014/471082

**Published:** 2014-04-13

**Authors:** Q. Q. Zhang, X. J. Wu, C. Wang, S. W. Zhu, Y. L. Wang, Bruce Z. Gao, X.-C. Yuan

**Affiliations:** ^1^Institute of Modern Optics, Key Laboratory of Optical Information Science & Technology, Ministry of Education of China, Nankai University, Tianjin 300071, China; ^2^Nankai University Affiliated Hospital, Tianjin 300121, China; ^3^Department of Bioengineering and COMSET, Clemson University, Clemson, SC 29634, USA; ^4^Institute of Micro & Nano Optics, Shenzhen University, Shenzhen 518060, China

## Abstract

Differences in tissue density cause a variety of scattering coefficients. To quantify optical coherence tomography (OCT) images for diagnosis, the tissue's scattering coefficient is estimated by curve fitting the OCT signals to a confocal single backscattering mode. The results from a group of 30 mice show that the scattering coefficients of bone, skin, liver, brain, testis, and spleen can be categorized into three groups: a scattering coefficient between 1.947 and 2.134 mm^−1^: bone and skin; a scattering coefficient between 1.303 and 1.461 mm^−1^: liver and brain; a scattering coefficient between 0.523 and 0.634 mm^−1^: testis and spleen. The results indicate that the scattering coefficient is tissue specific and could be used in tissue diagnosis.

## 1. Introduction

In spectral domain optical coherence tomography (SDOCT), a spectrometer is used to obtain high-resolution structural imaging through low coherence interferometry. The technique, which measures backscattered light generated by tissue, has great potential in disease diagnosis, including differentiation between benign and malignant lesions [1]. OCT typically employs near-infrared light to acquire micron-scale cross-sectional images of biological samples [2]. Both normal and premalignant tissues in various organs have been imaged by OCTs. For example, ovarian cysts, masses, and abnormal tissues have been successfully imaged using a laparoscopic OCT [3]. Laryngeal dysplasia and malignancy have been imaged with OCT probes during laryngoscopy [4], and malignant and inflammatory lymph nodes have been differentiated by OCT [5]. To gain more diagnostic information such as distinction between normal and abnormal tissues (as in histological analysis), OCT images must be further analyzed [6–10]. In addition to providing high-resolution morphological images, OCT can quantitatively provide the scattering coefficient (*μ*_*s*_). *μ*_*s*_ is approximately the total attenuation coefficient (*μ*_*t*_), which is the summation of *μ*_*s*_ and the negligible absorption coefficient (*μ*_*a*_) and can be estimated by fitting the OCT A-scan data to a theoretical model [11–13]. Using a single parameter such as *μ*_*t*_ to characterize in vivo tissue (instead of reconstructing and subsequently analyzing an OCT image), we may take the advantage of clinical OCT's high speed in vivo scanning [14, 15] and high spatial resolution detection [16] to achieve real-time clinical diagnoses. In the study, we use quantitative spectral domain optical coherence tomography to explore the scattering coefficients of various tissues or organs. Our approach is to take the OCT data as a function of depth with *μ*_*t*_ as a characteristic parameter and then fit the OCT data obtained from an actual tissue to the function to determine *μ*_*s*_ [6–10].

## 2. Principle and Methods

### 2.1. SDOCT System

**Figure 1:**
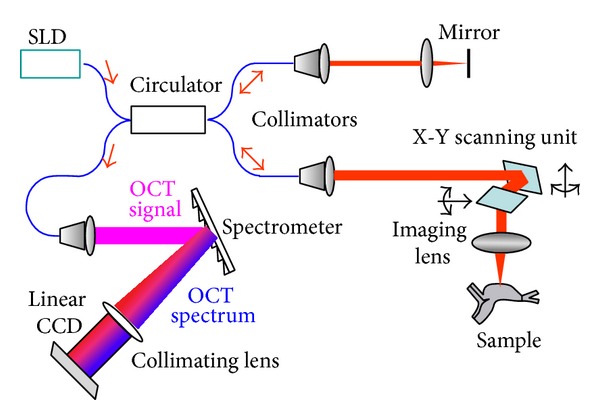
Schematic drawing of the SDOCT system.

The SDOCT system used in this study is described in our previous publication [17], and a schematic of the setup is shown in Figure 1. Light from a superluminescent diode (SLD) (S-840-B-I-20) with a bandwidth of 50 nm is split by a 50 : 50 fiber coupler into reference and sample arms. In the reference arm, the collimated beam from the single-mode fiber is reflected back from the reference mirror. In the sample arm, the collimated beam from the single-mode fiber is *x*-*y* scanned before being focused into the sample by a focusing lens (*f* = 35 mm). The OCT signal formed through interference of the reference and sample beams is detected by a custom spectrometer that consists of a fiber collimator, a diffraction grating (1200 lines/mm), an achromatic lens, and a line-scanning CCD (e2v AViiVA SM2 CL, 4096 × 1, 10 *μ*m). The resultant OCT spectrum is recorded by a linear CCD camera and transferred to the computer to implement fast Fourier transform by the CCD's camera link. In our system, the axial resolution, determined by the bandwidth of the light source, is approximately 6.2 *μ*m, and the lateral resolution, determined by the imaging system, is approximately 9.5 *μ*m.

The SDOCT imaging size is 2048 axial × 600 transverse pixels. To avoid a mirror image, light was focused on the surface of the scattering medium during imaging.

### 2.2. OCT Model

Two models are used for an OCT signal [11–13, 18–22]: the single-backscattering model and the multiple-scattering model. In the former, the light that has been backscattered only once contributes to the OCT signal, and the sample light is given by Beer's law:

(1) $$\begin{array}{c} I\left(z\right)\propto \exp\left(-2\mu_{t}z\right), \end{array}$$

where *I*(*z*) is the intensity of the interference signal; the factor 2 is due to the round-trip attenuation. The conventional single-backscattering model, which is suitable for dynamic focusing, ignores the coherence gate-generated confocal effect. To account for this confocal effect to establish a more accurate model, an axial confocal PSF for the OCT system was introduced by Faber et al. [12]. The OCT signal containing the confocal PSF is given as follows:

(2) $$\begin{array}{c} I\left(z\right)\propto \frac{\exp\left(-2\mu_{t}z\right)}{{\left(\left(z-z_{cf}\right)/z_{R}\right)}^{2}+1}, \end{array}$$

where *z*_*cf*_ is the position of the focal plane and *z*_*R*_ is the “apparent” Rayleigh length.

In our system, the numerical aperture of the imaging lens is 0.08, which is low for a fixed-focus OCT system [10], but provides sufficient focal zone for superficial scanning depth. The tissues we study are weakly scattering media (*μ*_*s*_ < 8 mm^−1^), so the single-backscattering model can be used in the OCT signal analysis to estimate the scattering coefficient [12, 23]. In our research, *μ*_*t*_ in (2) was replaced by *μ*_*s*_.

## 3. Quantitative Analysis of Mouse Organs 

**Figure 2:**
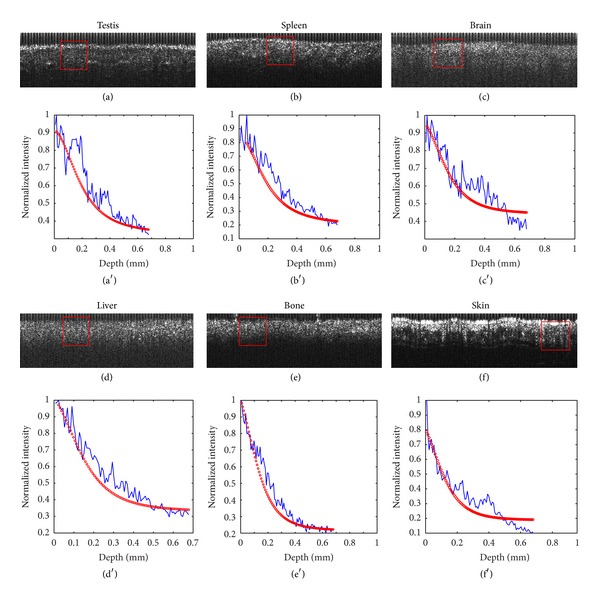
OCT images (a)–(f) and the corresponding curve fitting (a′)–(f′).

The study protocol for animal experimentation was approved by the Animal Protection Committee. Thirty identical BALB/c mice were used to obtain bone, skin, liver, brain, testis, and spleen. These organs from each mouse were washed by immersion in 0.9% saline solution and placed on the objective stage for imaging. Based on the acquired SDOCT signals, we used the OCT model to study scattering coefficients in different organs. Depending on the size of each sample, we performed 10–20 measurements at evenly distributed locations. Each measurement included 600 A-scans (each A-scan consisted of 4096 points) in which 3 evenly distributed regions of interest (ROIs), each consisting of 100 A-scans, were selected, as shown in Figure 2. A-scans in each ROI were averaged to estimate a scattering coefficient value. For each organ, we obtained a total of approximately 3 (ROIs) × 15 (measurements/sample) × 30 (samples) = 1350 scattering coefficient values for statistical analysis. To avoid surface effects, the signals from 20 *μ*m below the tissue surface were used for curving fitting.

Figure 2 shows OCT images (600 × 200 pixels) and the corresponding curve fittings; a typical image is selected for each organ. The red boxes highlight the 100 A-scans used to obtain an averaged A-scan for curve fitting. The differences in organ internal microstructures qualitatively reflected by the OCT images were quantified by the estimated *μ*_*s*_ values from Figures 2(a) to 2(f): 0.520 mm^−1^, 0.639 mm^−1^, 1.310 mm^−1^, 1.469 mm^−1^, 1.944 mm^−1^, and 2.130 mm^−1^, respectively.

**Figure 3:**
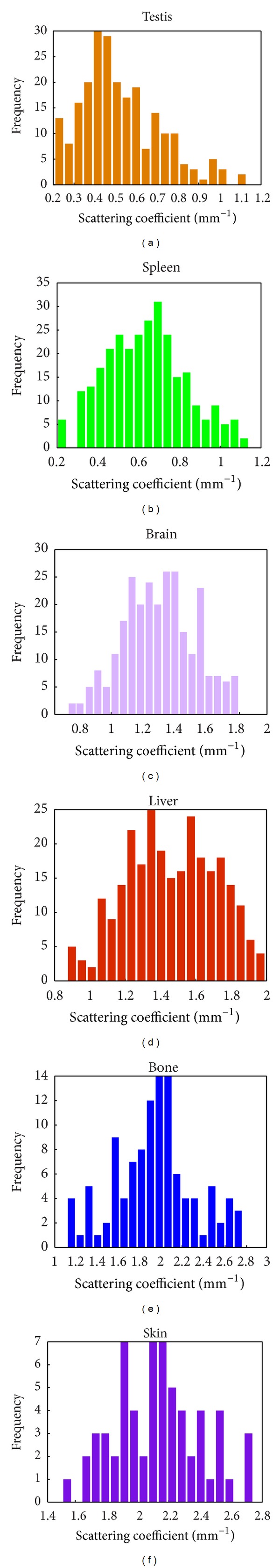
The histograms of each organ.

Figure 3 shows the histograms of *μ*_*s*_ values for each organ from the total measurements after discarding the maximum and minimum. Figure 3 shows that the distribution of *μ*_*s*_ conforms to a normal distribution, which is statistically meaningful. Because complete hair removal was difficult, the total number of OCT images for the skin is less than that for the other organs. However, the distribution of *μ*_*s*_ for the skin still conforms to a normal distribution.

The distribution of scattering coefficient *μ*_*s*_ is summarized in Table 1. Testis and spleen have smaller scattering coefficients. Brain and liver have larger scattering coefficients. Bone and skin have the largest scattering coefficients.

**Figure 4:**
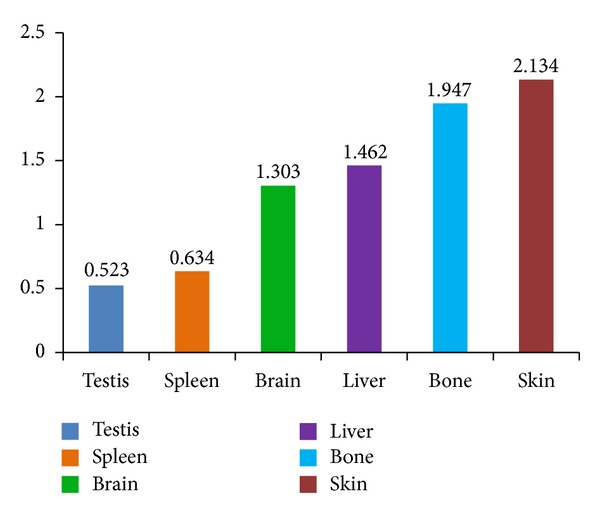
The distribution trend of organ scattering coefficients.

The data shown in Table 1 are presented as a bar graph in Figure 4. It shows that three groups in terms of the *μ*_*s*_ values can be classified: bone and skin, the high-value group; liver and brain, the intermediate-value group; and testis and spleen, the low-value group. As we know, bone and skin protect and support the body, preventing invasion of harmful substances. These organs share similar scattering coefficients because of their analogous structure. Testis, the male reproductive organ, contains contorted seminiferous tubules, and the spleen, the largest lymphoid organ in the body, is a reticular endothelial cell organ. This similar internal reticular structure places testis and spleen in the same class. The structures of the liver and brain are neither as dense as bone and skin, nor as loose as spleen and testis; thus, their scattering coefficients are between the scattering coefficients of those two groups.

This exploratory work in quantitative SDOCT indicates that each tissue and each organ have a unique basic value of *μ*_*s*_, which appears to be an identity value of its own. With this technology, the tissue can be recognized easily without professional knowledge of tissue-image segmentation. With the endoscopic technique, quantitative SDOCT can be used as a rapid and accurate tool for issue diagnoses.

## 4. Summary

In this study, we used the scattering coefficient to quantify tissues from mouse organs (bone, skin, liver, brain, testis, and spleen). The results show that these tissues can be roughly divided into three groups. The bone and skin group has mean scattering coefficients that are 1.947 and 2.134 mm^−1^, respectively; the liver and brain group has mean scattering coefficients of 1.303 and 1.461 mm^−1^; the testis and spleen group has mean scattering coefficients between 0.523 and 0.634 mm^−1^. SDOCT is a quantitative technology that is useful for basic tissue research and clinical diagnosis.

## Figures and Tables

**Table 1:** Distribution of scattering coefficients.

Organs	Range of scattering coefficient *µ*_*s*_ (mm^−1^)	Mean scattering coefficient *µ*_*s*_ (mm^−1^)
Testis	0.206–1.130	0.523 ± 0.036
Spleen	0.201–1.141	0.634 ± 0.038
Brain	0.729–1.797	1.303 ± 0.050
Liver	0.870–1.994	1.462 ± 0.064
Bone	1.130–2.763	1.947 ± 0.088
Skin	1.517–2.742	2.134 ± 0.079
